# Psychometric evaluation of the Osteoporosis Patient Treatment Satisfaction Questionnaire (OPSAT-Q™), a novel measure to assess satisfaction with bisphosphonate treatment in postmenopausal women

**DOI:** 10.1186/1477-7525-4-42

**Published:** 2006-07-11

**Authors:** Emuella M Flood, Kathleen M Beusterien, Hannah Green, Richard Shikiar, Robert W Baran, Mayur M Amonkar, David Cella

**Affiliations:** 1 United BioSource Corporation, Center for Health Outcomes Research, 7101 Wisconsin Avenue, Suite 600, Bethesda, MD 20814, USA; 2 Formerly of Roche Laboratories, Nutley, NJ, USA; 3 GlaxoSmithKline, Collegeville, PA, USA; 4 Evanston Northwestern Healthcare, Center on Outcomes Research and Education, Evanston, IL, USA; 5 Oxford Outcomes Inc., Bethesda, MD, USA

## Abstract

**Background:**

The Osteoporosis Patient Satisfaction Questionnaire (OPSAT-Q) is a new measure of patient satisfaction with bisphosphonate treatment for osteoporosis. The objective of this study was to evaluate the psychometric characteristics of the OPSAT-Q.

**Methods:**

The OPSAT-Q contains 16 items in four subscales: Convenience, Confidence with Daily Activities, Side Effects, and Overall Satisfaction. All four subscale scores and an overall composite satisfaction score (CSS) can be computed. The OPSAT-Q, Osteoporosis Targeted Quality of Life (OPTQoL), and sociodemographic/clinical questionnaires, including 3 global items on convenience, functioning and side effects, were self-administered to women with osteoporosis or osteopenia recruited from four US clinics. Analyses included item and scale performance, internal consistency reliability, reproducibility, and construct validity. Reproducibility was measured using the intraclass correlation coefficient (ICC) via a follow-up questionnaire completed by participants 2 weeks post baseline.

**Results:**

104 women with a mean age of 65.1 years participated. The majority were Caucasian (64.4%), living with someone (74%), and not currently employed (58.7%). 73% had osteoporosis and 27% had osteopenia. 80% were taking weekly bisphosphonates and 18% were taking daily medication (2% missing data). On a scale of 0–100, individual patient subscale scores ranged from 17 to 100 and CSS scores ranged from 44 to 100. All scores showed acceptable internal consistency reliability (Cronbach's alpha > 0.70) (range 0.72 to 0.89). Reproducibility ranged from 0.62 (Daily Activities) to 0.79 (Side Effects) for the subscales; reproducibility for the CSS was 0.81. Significant correlations were found between the OPSAT-Q subscales and conceptually similar global measures (p < 0.001).

**Conclusion:**

The findings from this study confirm the validity and reliability of the OPSAT-Q and support the proposed composition of four subscales and a composite score. They also support the use of the OPSAT-Q to examine the impact of bisphosphonate dosing frequency on patient satisfaction.

## Background

Osteoporosis is a major public health threat for 44 million Americans, 68% of whom are women [1]. Ten million individuals currently have osteoporosis in the United States and an additional 34 million have low bone mass or osteopenia. With osteoporosis responsible for over 1.5 million fractures annually, national direct expenditures (hospital and nursing home care) are estimated at approximately $14 billion each year [1].

The most commonly prescribed medications for osteoporosis are oral bisphosphonate treatments, such as ibandronate, alendronate or risedronate [2]. Other treatments include selective estrogen receptor modulators (SERMs) and hormone replacement therapy (HRT), calcitonin and the human recombinant parathyroid hormone active fragment (teraparatide). As is the case with many classes of chronically administered drugs, non-compliance with osteoporosis medications is a concern. Major reasons for non-compliance with osteoporosis treatment have been shown to include: fear of side effects, high drug prices, and inconvenience of drug regimens [3]. The association of medication compliance with frequency and convenience of bisphosphonate regimens has been explored in recent publications [4-6]. Regimens with less frequent dosing may contribute to perceptions of convenience and result in improved compliance.

Given the asymptomatic nature of osteoporosis and the absence of easily demonstrable treatment efficacy markers, along with the side effects and inconvenience associated with treatment, validated measures of patient satisfaction with osteoporosis medication hold great potential for evaluating current treatments from the patient's perspective and possibly for predicting patient adherence to treatment regimens. Treatment satisfaction is an increasingly important component of assessing overall quality of health care services and treatments, and satisfaction-related feedback from patients can be used to improve upon products released into the market [7]. Satisfaction with treatment has been shown to be associated with a desire to continue therapy [8].

Few satisfaction instruments have been developed in a rigorous manner based on patient concerns [9,10]. The OPSAT-Q™ (Appendix A [see Additional file 1]), a new measure of patient satisfaction with osteoporosis treatment, was developed in response to the increasing interest in patient-reported satisfaction with medication regimens and to meet the specific needs of assessing bisphosphonate treatments for osteoporosis. The objective of the current study was to evaluate the psychometric properties of the OPSAT-Q and, therefore, substantiate its use for patient satisfaction evaluation in studies of bisphosphonate treatment for osteoporosis and osteopenia.

## Methods

### OPSAT-Q development

The OPSAT-Q was recently developed based on a literature review and information attained from two focus groups. The literature review and focus groups were conducted to explore the impact of osteoporosis and its treatment on patient perceptions and to identify treatment attributes influencing patient satisfaction. The two focus groups were composed of women with osteoporosis or osteopenia taking daily or weekly bisphosphonates. Study participants were recruited through an advertisement in the Washington Post. The 18 focus group participants (10 in one group, 8 in the other) were primarily Caucasian (94%), had a mean age of 63.9 years, and the majority were taking alendronate (78%). One-third (33%) reported having been diagnosed with osteoporosis, with the remainder reporting a diagnosis of osteopenia.

The focus group discussions involved asking the participants to discuss the impact of osteoporosis/osteopenia and its treatment on their lives and the factors associated with treatment satisfaction. When asked what features of treatment are most important, the participants mentioned effectiveness ("observable results"; "that the progression of the disease is stopped"), not having side effects, cost, and convenience issues ("that you don't have to take it often") ("that it's not invasive"). Complaints regarding treatment included the cost, difficulty remembering to take the medication, the packaging, lack of "progress [observable improvement]," not knowing whether the medication is working, and side effects.

The data from the focus groups indicated that the following concepts are important to patients and directly influence patient satisfaction with bisphosphonate treatment: effectiveness, convenience issues, confidence with activities, side effects, and cost. Based on these findings, a draft osteoporosis patient treatment satisfaction questionnaire was developed. The draft questionnaire contained 21 items covering efficacy/effectiveness, convenience issues, confidence with activities, and side effects. Items assessing cost and packaging were not included because these are not applicable in a clinical trial setting. In addition, items on overall satisfaction were added. Comprehension of the draft questionnaire was tested through cognitive debriefing interviews with 15 additional women with osteoporosis or osteopenia taking daily or weekly bisphosphonates. The interviews involved asking the participants for their thoughts and opinions about phrasing, format, and content of the instrument. Results of the interviews showed that women with osteoporosis or osteopenia had difficulty with the efficacy/effectiveness items. Specifically, the respondents require physician feedback with respect to gauging efficacy/effectiveness outcomes and find it difficult to respond to questions on medication efficacy without this information, which is typically only received on an annual or bi-annual basis. Based on the feedback from the cognitive debriefings, items on efficacy and two side effects were removed from the instrument. The OPSAT-Q was revised and finalized for the psychometric evaluation study.

The OPSAT-Q (Appendix A [see Additional file 1]) contains 16 items comprising four domains: convenience (six items), confidence with daily functioning (two items), overall satisfaction (two items) and side effects (six items). Convenience, confidence with daily functioning, and overall satisfaction items are rated on a 7-point Likert-type scale from "very dissatisfied" to "very satisfied." Side effects include "heartburn/acid reflux," "stomach upset" and "other side effects" and are assessed in terms of both bother and frequency rated on a 5-point bother scale from "not at all bothered" to "extremely bothered" and a 5-point frequency scale from "0" to "> 3 days", respectively. All items were scored such that higher scores represented greater satisfaction (for satisfaction items) or less bother or frequency of side effects (for side effects items). Items 11–16 are reverse scored.

With respect to scoring, the items were *a priori *grouped into scales by their respective domains. These domain (scale) scores are calculated by summing the completed items in the domain and transforming the score to a 0 to 100 scale (sum of scores in domain/total possible score for domain × 100), where higher scores are more favorable. In addition, two scoring methods were proposed and tested as part of the psychometric evaluation. The first weights each domain equally, whereas the second weights each item equally. The findings showed the measurement properties to be very similar for each method. The most substantial difference was in Cronbach's alpha, with the second method having a higher value (0.87 vs. 0.72). Given the higher reliability of the second method, we report the findings for this method only. For calculating the CSS, all of the OPSAT-Q items are summed and transformed to a 0 to 100 scale, in which higher scores indicate greater satisfaction.

### Psychometric evaluation

#### Participants and procedures

Women taking bisphosphonates for osteoporosis or osteopenia were recruited from four US clinical sites, including primary care and rheumatology specialist clinics. The target sample size was 100 patients. The minimum sample size required for estimating correlations above 0.40 at an alpha level of 0.45 and 80% power is 47 patients [11]. Given that multiple correlations were to be preformed, we estimated that 100 patients would be an acceptable sample size. To be eligible for the study, participants had to be post-menopausal females with osteoporosis or osteopenia and currently using daily or weekly bisphosphonate treatment. Those with any concurrent medical condition or other impairment that, in the investigator's opinion, would preclude participation in this study were excluded. The sites were encouraged to enroll both daily and weekly bisphosphonate users.

The study protocol was approved by the Essex Institutional Review Board (Lebanon, NJ). Prior to study entry, all subjects provided written informed consent. All subjects completed a paper-and-pencil self-administered questionnaire at a baseline visit. The baseline questionnaire included the OPSAT-Q, Osteoporosis-Targeted Quality of Life Questionnaire (OPTQoL), and a Patient-Completed Demographic/Clinical questionnaire. The Patient-Completed Demographic/Clinical Questionnaire was used to characterize the study population. Associations between OPSAT-Q scores and OPTQoL scores, as well as selected demographic and clinical variables, were evaluated. A sub-sample of the first 50 willing subjects who completed the OPSAT-Q and a Follow-up Visit Clinical Questionnaire 1 to 2 weeks post-baseline was chosen to assess test-retest reliability of the OPSAT-Q.

### Study measures in addition to the OPSAT-Q

#### OPTQoL

The OPTQoL is a validated instrument used to assess the impact of osteoporosis on a patient's quality of life [12,13]. For the psychometric evaluation study of the OPSAT-Q, the OPTQoL was administered, which includes 5 domains, the following three of which are scored: Physical Difficulty, Adjustments, and Fears. The Physical Difficulty domain contains seven items rated on a 5-point Likert scale from "none" to "can't do it anymore." The Adjustments domain contains nine items and the Fears domain six items, each of which are rated on a 4-point Likert scale from "strongly disagree/no, not at all" to "strongly agree/a lot." The scores for each domain were converted to a 0 to 100 scale, in which higher scores indicate better quality of life.

#### Patient-reported demographic/clinical questionnaire

Participants completed a brief questionnaire on socio-demographic and clinical characteristics. The socio-demographic section included questions on age, ethnicity, living situation, employment, education, insurance status, and current medical conditions. Clinical questions included the date of osteoporosis/osteopenia diagnosis, current osteoporosis treatment, and 3 global questions on convenience, functioning and side effects.

#### Follow-up visit clinical questionnaire

The Follow-up Clinical Questionnaire was completed by the subsample of patients who returned for a follow-up visit. This questionnaire was used to identify any changes in clinical status since the baseline visit and included items assessing changes in health and osteoporosis treatment.

### Statistical analysis

Descriptive statistics were used to summarize the demographic and clinical characteristics of the study population at baseline. The analysis of the OPSAT-Q data focused on how well the items satisfied assumptions underlying Likert's method for summated ratings, which was used to score the OPSAT-Q scales. Score distributions, item-scale correlations, internal consistency reliability, reproducibility, and construct validity were assessed.

The distributions of the individual OPSAT-Q item and scale scores were examined by calculating the mean, median, minimum (least favorable or floor effect) and maximum (most favorable or ceiling effect) scores, and percent missing. Item to subscale correlations (Pearson product moment) also were evaluated. Item internal consistency is supported when all items in the same hypothesized scale are substantially correlated with each other (r > 0.40) [14].

Subscale to subscale correlations were also evaluated. The validity of scales are substantiated when conceptually related scales are substantially correlated with each other (r > 0.40).

### Reliability

Internal consistency reliability (Cronbach's alpha) was calculated for each of the OPSAT-Q scales. Minimum values of greater than or equal to 0.70 have been recommended for group level comparisons [15]. In addition, the reproducibility of the OPSAT-Q scales was assessed using both Pearson's and intra-class correlation coefficient estimates among stable participants over a 1 to 2 week period. Stable participants were defined as those who responded "no" to the following three questions on the Follow-up Questionnaire: 'Have you experienced a fracture since your last study visit?'; 'Have you experienced any side effects from your osteoporosis medication since your last study visit?'; and 'Have you had any changes in your osteoporosis medication since your last visit?'.

### Construct validity

Correlations between the OPSAT-Q scale scores and the three global items (in the Demographic/Clinical Questionnaire) focusing on overall convenience, fear of performing activities, and side effects, as well as the three OPTQoL subscales, were evaluated at baseline. Concurrent validity was supported when a specific scale was substantially correlated (> 0.40) with a conceptually-related scale. The ability of the scores to discriminate between groups of patients according to dosing frequency (daily vs. weekly bisphosphonates), diagnosis (osteopenia vs. osteoporosis), and history of fracture since menopause was assessed using t-tests.

## Results

### Sample characteristics

A total of 104 patients participated in the study. Patient characteristics of the enrollment sample are summarized in Table 1. The average age of the sample was 65.1 years. The majority were Caucasian (64.4%), living with someone (74%), and not currently employed (58.7%). With respect to diagnosis, 73% were diagnosed with osteoporosis and the remaining 27% with osteopenia. Mean time since diagnosis of either osteoporosis or osteopenia was 6.4 years. The most common comorbidities reported were arthritis (57%) and hypertension (31%). Sixteen patients (15.4%) had no health insurance.

**Table 1:** Demographic and Clinical Characteristics

**Item**	**Enrolled (N = 104)**
**Age (years) mean (SD)**	65.1 (10.3)
**Race n (%)**	
White	67 (64.4%)
Black	1 (1.0%)
Hispanic	36 (34.6%)
**Living Situation**	
Living alone	25 (24.0%)
Living with another	77 (74.0%)
Other	2 (1.9%)
**Work Status**	
Full-time	17 (16.3%)
Part-time	19 (18.3%)
Currently not working at a paid job	61 (58.7%)
Other	7 (6.7%)
**Education **(highest level completed)	
Elementary	4 (3.8%)
High school	38 (36.5%)
Some college	31 (29.8%)
College degree	19 (18.3%)
Postgraduate	10 (9.6%)
Other	2 (1.9%)
**Time since diagnosis **(years), mean ± SD	6.4 ± 7.0
**No Health Insurance**	16 (15.4%)
**Time since first starting prescription meds for osteoporosis (bisphosphonates)**	
< 3 months ago	7 (6.7%)
> 3 months – 1 year	14 (13.5%)
> 1 – 3 years ago	35 (33.7%)
> 3 years	48 (46.2%)
**Current Osteoporosis Treatment^1^**	
Weekly alendronate	61 (59%)
Daily alendronate	13 (13%)
Weekly risendronate	21 (20%)
Daily risendronate	6 (6%)
**Experienced at least 1 fracture since menopause **n (%)	27 (26.0%)
**Comorbid conditions **n (%)	
Angina	3 (2.9%)
Arthritis	59 (56.7%)
COPD	6 (5.8%)
Congestive heart failure	3 (2.9%)
Depression	17 (16.3%)
Hypertension	32 (30.8%)
Other	26 (25.0%)

^1^Missing medication data for 1 subject; missing medication frequency data for 2 risedronate subjects.

Nearly half the sample (46%) had started taking prescription medication for osteoporosis over 3 years earlier. All subjects reported taking bisphosphonates (72% alendronate, 28% risedronate) and most (80%) were taking them on a weekly basis. The most common regimen was weekly alendronate (59%), followed by weekly risedronate (20%), daily alendronate (13%), and daily risedronate (6%). Dosing frequency data was missing for 2 subjects taking alendronate.

One subject was excluded from the analyses due to highly discrepant responses based on review of scatter plots. Thus, the psychometric analyses were based on a total sample of 103 subjects.

### Item and scale properties

Item and scale distributions for the OPSAT-Q were examined. With respect to items rated on the 7-point "satisfaction" response scale (items 1–10), where 1 reflects "very dissatisfied" and 7 reflects "very satisfied," mean item scores ranged from 5.7 (confidence to be physically active) to 6.2 (how often you take the medication). For the side effect items (items 11–16), which were rated on a 5-point Likert scale for bother (items 11–13) and frequency (items 14–16) (1 = not at all bothered/0 days; 5 = extremely bothered/>3 days), the mean values ranged from 4.2 (heartburn/acid reflux frequency) to 4.8 (stomach upset bother and other side effects bother).

Table 2 provides the subscale and CSS score distributional characteristics at baseline. On a scale of 0 to 100, mean subscale scores ranged from 81.1 (Daily Activities) to 89.6 (Side Effects). Daily Activities and Overall Satisfaction covered a range of 17–100, while Convenience and Side Effects covered a range from 31 to 100 and 42 to 100, respectively. The range for the CSS was 44 to 100. The percentage of subjects scoring at the floor was 1% for all subscales and composite scores. Among the subscales, the percentages of subjects scoring at the ceiling ranged from 10.7% for Convenience to 45.6% for Side Effects.

**Table 2:** OPSAT-Q™ Subscale Distributional Characteristics at Baseline (N = 103)

**Scale**	**Mean**	**SD**	**Median**	**Floor (%)**	**Ceiling (%)**	**Range**
Convenience	83.4	13.5	83.3	1.0%	10.7%	31–100
Daily Activities	81.1	16.9	83.3	1.0%	22.3%	17–100
Overall Satisfaction	84.1	15.7	83.3	1.0%	28.2%	17–100
Side Effects	89.6	14.3	95.8	1.0%	45.6%	42–100
Composite Satisfaction Score	84.9	10.6	86.9	1.0%	4.8%	44–100

Floor = percent who answered minimum value. Ceiling = percent who answered maximum value.

Table 3 reports item-to-scale Pearson product-moment correlations. As expected, all items within each subscale were more highly correlated with their hypothesized subscale relative to the other subscales, and all 16 items were significantly correlated with the CSS.

**Table 3:** OPSAT-Q™ Item-to-Subscale and CSS Correlations at Baseline^a ^(N = 103)

	**Subscale**				
**Item**	**Convenience**	**Daily Activities**	**Overall Satisfaction**	**Side Effects**	**CSS**
1 How often	0.78***	0.42***	0.54***	0.07	0.66***
2 Convenience	0.89***	0.39***	0.51***	0.17	0.75***
3 Easy to take	0.84***	0.40***	0.45***	0.13	0.70***
4 Easy to remember	0.66***	0.29**	0.25*	0.09	0.51***
5 Fits into medication schedule	0.83***	0.35***	0.49***	0.11	0.68***
6 Time required	0.85***	0.51***	0.51***	0.28**	0.80***
7 Confidence to participate in daily activities	0.46***	0.92***	0.69***	0.15	0.66***
8 Confidence to be physically active	0.44***	0.94***	0.68***	0.21*	0.68***
9 Overall satisfaction	0.52***	0.75***	0.95***	0.17	0.72***
10 Continue taking	0.54***	0.63***	0.93***	0.15	0.69***
11 Hearburn/acid reflux bother	0.29**	0.27**	0.25*	0.58***	0.49***
12 Other stomach upset bother	0.17	0.05	-0.06	0.63***	0.33***
13 Any other side effect bother	0.11	0.12	0.14	0.68***	0.38***
14 Heartburn/acid reflux freqency	0.13	0.16	0.21*	0.73***	0.42***
15 Other stomach upset frequency	-0.01	0.08	0.02	0.57***	0.24*
16 Any other side effect frequency	0.08	0.09	0.06	0.75***	0.37***

*<0.05, **<0.01, ***<0.001; ^a^Pearson product moment correlations; ^b^Convenience subscale includes items 1–6, Daily Activities subscale included items 7 and 8, Overall Satisfaction subscale includes items 9 and 100, and the Side Effects subscale includes items 11–16.

### Reliability

The internal consistency reliability estimates, reflected by Cronbach's alpha, for each subscale and CSS score at baseline are presented in Table 4 (recommended level = 0.70). Among the subscales, Cronbach's alpha ranged from 0.72 for Side Effects to 0.89 for Convenience. The CSS had a Cronbach's alpha of 0.87.

**Table 4:** OPSAT-Q™ Internal Consistency Reliability at Baseline (Cronbach's Alpha) and Reproducibility (N = 103)

**Scale**	**Number of Items**	**Cronbach's alpha**	**ICC^a^**
Convenience	6	0.89	0.72
Daily Activities	2	0.84	0.62
Overall Satisfaction	2	0.87	0.64
Side Effects	6	0.72	0.79
Composite Satisfaction Score	16	0.87	0.81

^a^Intra-class correlations between baseline and follow-up visit among stable patients.

Reproducibility was evaluated for patients considered stable (n = 46), meaning they did not report changes in health or osteoporosis medication at the follow-up visit. Based on review of scatter plots, three subjects were identified as extreme outliers and were excluded from the test-retest reliability analysis. Among the subscales, reproducibility based on Pearson's and intra-class correlation coefficients ranged from 0.62 for Daily Activities to 0.79 for Side Effects. Reproducibility for the CSS was 0.81 (Table 4).

### Construct validity

Table 5 reports the correlations between each of the three global items (overall convenience, overall fear, and overall side effects) and the three OPTQoL subscales with the OPSAT-Q scale and CSS (substantial correlations ≥ 0.4). As hypothesized, Overall Convenience was most highly correlated with the OPSAT-Q Convenience scale (0.73; p < 0.001) relative to the other OPSAT-Q subscales. Similarly, Overall Fear was most highly correlated with Daily Activities (0.43; p < 0.001) relative to the other OPSAT-Q subscales. Finally, Overall Side Effects was most highly correlated with the Side Effects scale (0.57; p < 0.001) relative to the other OPSAT-Q subscales. All three global items were significantly associated with the CSS.

**Table 5:** Construct Validity at Baseline: Correlation between OPSAT-Q™ and Global Items and OPTQoL Scores

	**OPSAT-Q™ Scales and Composite Satisfaction Score (CSS)**				
	**Convenience**	**Daily Activities**	**Side Effects**	**Overall Satisfaction**	**CSS**
Overall Convenience	0.73***	0.47***	0.29**	0.58***	0.74***
Overall Fear	0.22*	0.43***	0.15	0.42***	0.37***
Overall Side Effects	0.27**	0.29**	0.57***	0.24*	0.48***
OPTQoL Adjustments	0.37***	0.33***	0.23*	0.21*	0.40***
OPTQoL Physical Difficulty	0.17	0.27**	0.10	0.17	0.23*
OPTQoL Fears	0.16	0.32***	0.32***	0.15	0.32**

^1^Pearson product moment correlations or Spearman-rank correlations. *<0.05, **<0.01, ***<0.001

Correlations between the three OPTQoL subscales and the OPSAT-Q scales also are reported in Table 5. A review of the content of the OPTQoL items shows that all three scales focus in some way on performing activities. Specifically, the OPTQoL Physical Difficulty scale focuses on having difficulties performing certain activities, the OPTQoL Adjustments scale focuses on planning certain activities, and the OPTQoL Fears scale focuses on fears of sustaining pain or injuries while performing certain activities. Consistent with their highly "physical" focus, all three OPTQoL scales were significantly correlated with the OPSAT-Q Daily Activities scale (p < 0.01). Of the three OPTQoL scales, the OPSAT-Q Convenience scale was significantly correlated with the Adjustments scale (0.37; p < 0.001), which may be expected because the Adjustments scale focuses on planning, which in some ways is related to Convenience. Finally, the OPSAT-Q Side Effects scale was most highly correlated with the OPTQoL Fears scale, which focuses on fear of sustaining pain and injuries while performing certain activities (0.32; p < 0.001).

Comparisons of OPSAT-Q scores were also made between selected subgroups of participants: daily (n = 20) versus weekly (n = 83) administration; osteoporosis (n = 75) versus osteopenia (n = 28); and history of fracture (n = 26) versus no fracture history (n = 77). Although no differences between groups were significant, which in part may be attributable to sample sizes, the OPSAT-Q scales appeared to vary in their sensitivity to differences. For example, the Convenience and Overall Satisfaction scales showed greater mean differences favoring the weekly bisphosphonate users versus the daily users relative to the other scales. In addition, the Daily Activities scale showed a greater mean difference favoring osteopenia subjects versus those with osteoporosis relative to the other scales.

## Discussion

The findings from this US study substantiate evidence that the OPSAT-Q subscale scores are valid and that the subscale scores and composite score are reliable for measuring treatment satisfaction with bisphosphonates for osteoporosis and osteopenia among post-menopausal women. The OPSAT-Q subscales had acceptable measurement properties and the analyses supported the proposed scoring structure of the instrument, specifically the composition of four subscales: Convenience, Daily Activities, Overall Satisfaction, and Side Effects. They also suggest that using a composite score is acceptable. The subscale and composite scores were internally consistent and, although reproducibility was slightly lower than the recognized standard of 0.7 for two of the subscales (Daily Activities and Overall Satisfaction) (0.62, 0.64), the measurement properties were consistent with other treatment satisfaction measures [16-18]. The psychometric properties of the OPSAT-Q support its use in performing group level comparisons in future studies of bisphosphonate treatment.

The baseline OPSAT-Q scores indicated that subjects in this analysis were generally satisfied with their osteoporosis medication. This finding is consistent with the patients' overall length of time on their medications. Over 75% of patients had been taking bisphosphonates for greater than 1 year and 46% had been on medication greater than 3 years (Table 1), indicating that they had adapted particularly well to the regimens. Nevertheless, given the small to moderate ceiling effects observed for the subscale and composite scores, it appears that this measure can potentially capture improvements in satisfaction. It should be noted that satisfaction items tend to be skewed upward (i.e., more positive ratings of satisfaction) for treatment satisfaction measures [9,10,19]. The responses to the side effect questions were more highly skewed, but this may be related to the fact that only a subgroup of patients taking bisphosphonates experience side effects, and that this subgroup has a higher risk of having discontinued treatment. Also, the majority of the study patients had been on bisphosphonate medication for greater than one year; hence, it is possible that those patients who had more severe side effects when starting on the drug would have stopped taking the drug after a short period of time, and thus not presented themselves for this study of current bisphosphonate users. Alternatively, the patients who did present for the study may have increased their tolerance to side effects.

The composition of the subscales is supported by the fact that the OPSAT-Q items were more highly correlated with their hypothesized scales than competing scales. Construct validity of the OPSAT-Q was supported by the significant correlations between the subscales and similar global measures and quality-of-life scales. These correlations also supported the current domain structure of the instrument. And, although statistically significant differences in OPSAT-Q scores were not observed between selected subgroups of patients (daily vs. weekly bisphosphonate users, osteopenia vs. osteoporosis diagnosis, history of fracture vs. no history of fracture), the direction of the differences observed for selected scales would have been hypothesized given their focus.

One limitation of the study is that the focus groups comprised a sample of volunteers, which is not representative of all women with osteoporosis taking bisphosphonates in the US. Nevertheless, the study did include recruitment from four sites in different geographic regions in the US. Previous research has shown effectiveness to be an important component of treatment satisfaction [7]. In this study, effectiveness was not included as part of the OPSAT-Q, because based on the focus group findings, women with osteoporosis or osteopenia require physician feedback in order to gauge efficacy/effectiveness. Specifically, participants in the focus groups were generally unable to assess the efficacy/effectiveness of their medication in the absence of a BMD DXA measurement. Thus, participants were either unable to provide a meaningful response regarding effectiveness, or responded based upon their satisfaction with their most recent bone scan results. Also, sub-group sample sizes were relatively small for some of the discriminant validity analyses (e.g., comparing osteoporosis to osteopenia patients). Finally, a major reason for assessing patient satisfaction is that there are expected consequences as a result of differences in satisfaction, for example, in terms of adherence to prescription regimens and drug switching to alternative medications. It should be underlined that the design of this study was focused on assessing the psychometric properties of the OPSAT-Q. Assessment of its value in predicting important behaviors requires a longitudinal design, which was beyond the scope of the present study.

## Conclusion

The OPSAT-Q demonstrated acceptable measurement properties, including validity of the subscales and reliability of the subscale and composite scores. The findings support the use of the OPSAT-Q as a treatment satisfaction measure in clinical studies of bisphosphonate treatment for osteoporosis and osteopenia. The OPSAT-Q can be used to quantify enhanced satisfaction with improved bisphosphonate regimens. Future studies that include the OPSAT-Q can help to further substantiate the findings from this study.

## Abbreviations

CSS – Composite Satisfaction Score

HRT – Hormone Replacement Therapy

ICC – Intraclass Correlation Coefficient

OPSAT-Q – Osteoporosis Patient Satisfaction Questionnaire

OPTQoL – Osteoporosis Targeted Quality of Life

## Competing interests

Mayur Amonkar is an employee of GlaxoSmithKline while Robert Baran was an employee of Roche at the time this study was conducted. The remaining authors declare that they have no competing interests.

## Authors' contributions

EF drafted the manuscript and participated in the design, data collection, and data analysis. KB helped draft the manuscript and participated in the design and data analysis and interpretation of the findings. HG reviewed the manuscript and assisted in collecting and cleaning the data. RS reviewed the manuscript and participated in the study design, analysis and interpretation of the findings. RWB reviewed the manuscript, initiated the concept, and participated in the design and implementation of the study. MMA reviewed the manuscript and participated in the study concept and design and interpretation of the findings. DC reviewed the manuscript and participated in the study design, analysis and interpretation of findings. All authors read and approved the final manuscript.

## Supplementary Material

Additional File 1 Flood. Appendix A. OPSAT-Q™ Click here for file
