# Supplementary material for: Psychometric evaluation of the Osteoporosis Patient Treatment Satisfaction Questionnaire (OPSAT-Q™), a novel measure to assess satisfaction with bisphosphonate treatment in postmenopausal women
Source: Health Qual Life Outcomes. 2006 Jul 11;4:42. doi: 10.1186/1477-7525-4-42 (PMC1550233; doi:10.1186/1477-7525-4-42)
Supplement: Additional File 1 — Flood. Appendix A. OPSAT-Q™ [file 1477-7525-4-42-S1.doc]

**Appendix A. OPSAT-Q™**

The following questions ask about how satisfied or dissatisfied you are with the medication you have been taking for osteoporosis or osteopenia. Please answer each question by marking an “X” in the box that most closely represents the way you feel. There are no right or wrong answers.

Please indicate how ***satisfied or dissatisfied*** you are with the following…

|  | **Very**  **Dissatisfied** | **Dissatisfied** | **Somewhat Dissatisfied** | **Neither**  **Satisfied nor**  **Dissatisfied** | **Somewhat Satisfied** | **Satisfied** | **Very**  **Satisfied** |
| --- | --- | --- | --- | --- | --- | --- | --- |
| 1. **How often** you have to take the medication | □ | □ | □ | □ | □ | □ | □ |
| 2. The **convenience** of taking the medication | □ | □ | □ | □ | □ | □ | □ |
| 3. How **easy it is to take** the medication | □ | □ | □ | □ | □ | □ | □ |
| 4. How **easy it is to remember** to take the medication | □ | □ | □ | □ | □ | □ | □ |
| 5. How well the medication **fits into your overall medication schedule** | □ | □ | □ | □ | □ | □ | □ |
| 6. The amount of **time required** to take the medication, including staying upright | □ | □ | □ | □ | □ | □ | □ |
| 7. How well the medication gives you **confidence to participate in your daily home and/or work activities** | □ | □ | □ | □ | □ | □ | □ |
| 8. How well the medication gives you **confidence to be as physically active** as you’d like to be | □ | □ | □ | □ | □ | □ | □ |
| 9. **Overall,** **how satisfied are you** with your medication? | □ | □ | □ | □ | □ | □ | □ |
| 10. How satisfied would you be **to continue taking** the medication? | □ | □ | □ | □ | □ | □ | □ |

How **bothered** are you by the following **side effects** that you may or may not experience after taking your osteoporosis/osteopenia medication? If you have never experienced the side effect from the medication, please answer “Not at All Bothered.”

|  | **Not at All Bothered** | **Slightly**  **Bothered** | **Moderately**  **Bothered** | **Quite a Bit Bothered** | **Extremely Bothered** |
| --- | --- | --- | --- | --- | --- |
| 11. **Heartburn or acid reflux** | □ | □ | □ | □ | □ |
| 12. **Stomach upset other than heartburn or acid reflux** (such as diarrhea, nausea, vomiting, or stomach pain) | □ | □ | □ | □ | □ |
| 13. **Any other side effects** you think are related to your osteoporosis medication | □ | □ | □ | □ | □ |

During the past 4 weeks, on approximately **how many days** did you experience the following **side effects** associated with your osteoporosis/osteopenia medication?”

|  | **0 Days** | **1 Day** | **2 Days** | **3 Days** | **More than**  **3 Days** |
| --- | --- | --- | --- | --- | --- |
| 14. **Heartburn or acid reflux** | □ | □ | □ | □ | □ |
| 15. **Stomach upset other than heartburn or acid reflux** (such as diarrhea, nausea, vomiting, or stomach pain) | □ | □ | □ | □ | □ |
| 16. **Any other side effects** you think are related to your osteoporosis medication | □ | □ | □ | □ | □ |

© 2005 Roche Laboratories, Inc. All Rights Reserved.
